# CREB Regulates Experience-Dependent Spine Formation and Enlargement in Mouse Barrel Cortex

**DOI:** 10.1155/2015/651469

**Published:** 2015-05-05

**Authors:** Annabella Pignataro, Antonella Borreca, Martine Ammassari-Teule, Silvia Middei

**Affiliations:** ^1^Laboratory of Psychobiology, Santa Lucia Foundation, 00143 Rome, Italy; ^2^“Tor Vergata” University, 00173 Rome, Italy; ^3^Institute of Cell Biology and Neurobiology (IBCN), National Research Council, 00015 Rome, Italy

## Abstract

Experience modifies synaptic connectivity through processes that involve dendritic spine rearrangements in neuronal circuits. Although cAMP response element binding protein (CREB) has a key function in spines changes, its role in activity-dependent rearrangements in brain regions of rodents interacting with the surrounding environment has received little attention so far. Here we studied the effects of vibrissae trimming, a widely used model of sensory deprivation-induced cortical plasticity, on processes associated with dendritic spine rearrangements in the barrel cortex of a transgenic mouse model of CREB downregulation (mCREB mice). We found that sensory deprivation through prolonged whisker trimming leads to an increased number of thin spines in the layer V of related barrel cortex (*Contra*) in wild type but not mCREB mice. In the barrel field controlling spared whiskers (*Ipsi*), the same trimming protocol results in a CREB-dependent enlargement of dendritic spines. Last, we demonstrated that CREB regulates structural rearrangements of synapses that associate with dynamic changes of dendritic spines. Our findings suggest that CREB plays a key role in dendritic spine dynamics and synaptic circuits rearrangements that account for new brain connectivity in response to changes in the environment.

## 1. Introduction

To adapt to the surrounding environment, neural circuits rearrange by means of strengthening or weakening of their synapses. Modifications in connectivity are supported by variations in number and size of dendritic spines, small protrusions along dendritic branches that host excitatory postsynaptic sites.

Most spines formed in adult individuals are transient. In cortical regions of young adult rodents, the vast majority of newly formed spines shrink and disappear rapidly. Only a small minority (20%) of new spines persist over the 48 hours following their formation [1] and an even smaller proportion (3%) last up to 30 days [2, 3]. The occurrence of a synaptic contact, as identified by the presence of a postsynaptic density (PSD) opposed to an active synaptic zone [4], is a key determinant for the persistence of a spine. A common view emerging from these evidences is that the stabilization of a spine reflects the presence of a synapse, and therefore variations in number and/or size of spines regulate the amount of excitatory inputs within a neuronal circuit.

Interaction with the surrounding environment strongly affects the plasticity within cortical circuits. Both stimuli coming from the movements of vibrissae when rodents explore surrounding environments and sensory deprivation resulting from vibrissae deletion can trigger rearrangements in somatosensory cortex and constitute remarkable tools to study the dynamics of cortical spines and their relevance for circuits plasticity. In rodent's somatosensory cortex, barrel fields are topographic organizations receiving sensory information from contralateral whiskers via thalamocortical projections [5]. Modifications of sensory inputs induced through whisker trimming strongly alter spine stability with a variability that depends on the developmental age, the duration, and the experimental model of the trimming procedure. In 6- to 10-week-old mice, trimming whiskers for 3 to 4 days according to a chessboard pattern associates with a reduction of stable spines while increasing the proportion of transient ones in the corresponding barrel cortex [6]. In young adolescent (30 days old) mice, prolonged unilateral deprivation induced by cutting of all whiskers associates with a reduction of baseline spine elimination, thus resulting in a net increase in spine density on neurons of layer V contralateral barrel cortex [1]. Together, these evidences suggest that deprivation destabilizes old spines and favors new spine stabilization [3].

Understanding the intracellular pathways that participate in growth or retraction of spines in adulthood has a key relevance to contrast pathological conditions that manifest with either massive formation or excessive pruning of dendritic spines [7]. Among these pathways, the one involving cAMP response element binding protein (CREB) has been deeply investigated in relation to synaptogenesis [8] and new spine formation [9–11] since its discovery as a transcription factor involved in synaptic plasticity events [12, 13]. Despite this, few studies [14, 15] have focused on CREB regulation of adult spine formation in relation to external inputs so far. Although these papers clearly demonstrate that CREB is necessary for spine formation and shape modification, how the CREB-dependent spine dynamics are able to impact synaptic circuit connectivity has to be elucidated yet.

To answer this question, we investigated modifications of dendritic spines and related synaptic inputs in cortical circuits following prolonged unilateral whisker cut in young transgenic mice with CREB downregulation. Our results point to a key role of CREB in both spines dynamics and synaptic rearrangements in response to modifications in sensory experience.

## 2. Materials and Methods

### 2.1. Experimental Animals

The mCREB is a phosphorylation defective form of CREB in which a Serine to Alanine substitution prevents phosphorylation at Serine 133 site [16, 17]. Transgenic mCREB mice and littermates wild type used in this study were generated by crossing mice expressing the tetracycline transactivator (tTA) under the control of the *α*-calmodulin kinase II (*α*CaMKII) promoter (*α*CaMKII-tTA mouse line [18], Jackson Laboratories) with mice bearing the mCREB mutation under control of the tetracycline-operated promoter (tetOp-mCREB mouse line [19], kindly donated by R. Duman, Yale University) to provide a spatial and temporal control over transgene expression. Both lines were on C57BL/6J mice transgenic background. In double transgenic (mCREB) mice from F1 generation, the *α*CaMKII promoter spatially restricts mCREB expression to forebrain principal neurons and the administration of doxycycline (analogue of tetracycline) prevents the mCREB expression driven by tetracycline-operated promoter. To exclude any developmental alterations due to repression of CREB function, food containing 40 mg/kg doxycycline (Bio-Serv, Frenchtown, NJ, USA) was delivered to pregnant females during the last week of pregnancy and to all litters from birth to weaning (postnatal day (PND) 21). Upon weaning, removing doxycycline from the diet induced the mCREB expression. Experiments started at PND 30, that is, 10 days after transgene expression in mCREB mice. Mice were genotyped by PCR to detect the two transgenes. Single transgenic and wild type littermates were included in control wild type groups as in [14, 20]. Animals were housed in groups of 4 in transparent Plexiglas cages placed in an air-conditioned room (22°C) with light-dark 12/12 hours cycle and with food and water ad libitum. An investigator blind to the experimental conditions of the groups performed all experiments. Experiments were conducted in accordance with the guidelines laid down by the European Communities Council Directive (86/609/EEC). The study received institutional approval (D.M. number 215/2010B).

### 2.2. Whisker Cut Protocol and Experimental Groups

**Figure 1:**
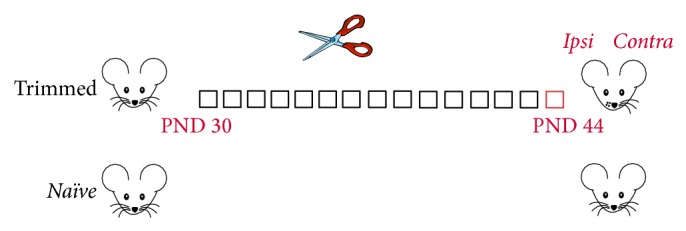
Whisker trimming procedure. Cartoon depicting the whisker trimming protocol. Wild type and mCREB mice underwent 14 days of unilateral whisker deprivation starting at 30 days of age or left undisturbed in their home cage (*Naïve*). PND: postnatal day. At the end of trimming period, brains were separated and the two hemispheres were identified as ipsilateral (*Ipsi*) or contralateral (*Contra*) to trimmed mystacial pad.

mCREB and wild type mice were whisker trimmed starting from PND 30 and sacrificed at PND 44. Whisker trimming was performed daily for two weeks by cutting the mystacial vibrissae to skin level with surgical scissors (Figure 1). A separate group of mCREB and wild type mice were left undisturbed in the home cage and scarified at PND 44 (*Naïve* mice). From each brain of whisker-trimmed mice, the two hemispheres were separated and included in the* Contra* group (hemisphere contralateral to the trimming side) and* Ipsi* group (hemisphere ipsilateral to the trimming side). Brains of* Naïve* mice were collected and included in the* Naïve* group. Five to six mice from each experimental group were sacrificed with saline perfusion and brains were collected for Golgi staining. Six mice per experimental group were perfused with paraformaldehyde (4%) for immunofluorescence studies.

### 2.3. Golgi Cox Staining and Dendritic Spine Analysis

Brain samples of wild type and mCREB mice from all experimental groups were collected and processed for Golgi-Cox staining. Mice were perfused with saline 0.9% and samples were impregnated in a Golgi-Cox solution (1% potassium dichromate, 1% mercuric chloride, and 0.8% potassium chromate) at room temperature according to a previously described protocol [21]. Six days after, the samples were placed in sucrose (30%) for three days and sectioned coronally (100 *μ*m) using a vibratome. Sections were stained through consecutive steps in water (1 minute), ammonium hydroxide (30 minutes), water (1 minute), developer solution (Kodak fix 100%, 30 minutes), and water (1 minute). Sections were then dehydrated through successive steps in alcohol at rising concentrations (50%, 75%, 95%, and 100%) before being closed with slide cover slips. Spine analysis was performed on 20 *μ*m dendritic segments of basal and apical dendrites of pyramidal neurons having their cell bodies in layer V of barrel cortex. For basal dendrites, the analysis was restricted to layer V only by selecting segments that (i) were lying along second to fourth order dendrites, (ii) run horizontally to the cell body, and (iii) were distant at least 150 *μ*m from the cell body (see details in Figure 3(a)). For apical dendrites, spine analysis was performed on second and third order dendrites lying along IV to II cortical layers. Spine density was measured online using the software Neurolucida (Microbrightfield) connected to an optical microscope DMLB Leika. Analysis of dendritic spines size was carried out on random selection of counted spines by measuring spine width parallel to dendrite through the ImageJ software (NIH, USA) according to procedures previously described [22]. Spine head width values were expressed as cumulative frequencies and compared among groups.

### 2.4. Immunofluorescence

Brains were removed immediately after perfusion and postfixed in 4% paraformaldehyde overnight at 4°C. The day after, brains were cryoprotected in 30% sucrose in PBS. Coronal sections of 40 *μ*m were prepared for immunofluorescence. Before incubation with primary antibodies, slices were washed in cold methanol (10 minutes, methanol 100%) and then further washed in PBS 1X to improve the detection of PSD 95 signal (as recommended in http://www.cellsignal.com/contents/resources-protocols/immunofluorescence-protocol-with-methanol-permeabilization-%28if-methanol-perm%29/if-methanol). Brain slices were then incubated with primary antibodies in PBS 1X, 3% triton, at 4°C. Primary antibodies used were to PSD 95 (Anti-Rabbit, 1 : 100, o/we; Abcam), vesicular glutamate transporter 1 (vGlut1 Anti-Guinea Pig, 1 : 800, o/we; Synaptic System), phosphorylated CREB (pCREB, Anti-Rabbit, 1 : 1000, o/n; Millipore), and CREB (CREB, Anti-Rabbit, 1 : 600, o/n; Cell Signaling). Slices were then washed three times with PBS 1X and incubated with appropriate fluorescently labelled secondary antibodies (room temperature, dark, 2 hours). Slices were further washed three times in PBS 1X and then were counterstained with DAPI (1 : 1000, 10 minutes; Enzo life Science) after the final PBS wash. At last, sections were mounted with Fluoromount (Sigma Aldrich) and cover-slipped.

### 2.5. Image Analysis

Images from* Ipsi*,* Contra*, and* Naïve* samples were acquired with a confocal microscope (Zeiss LSM700). Layer V barrel cortex was first identified under low magnification (10x). Subsequently, images for pCREB and CREB signals were acquired at 20x/zoom1x magnification; images for PSD 95 and vGlut1 signals were acquired at 100x magnification. Two to four images were acquired from each identified hemisphere (*Contra*,* Ipsi*) in trimmed groups or from the two hemispheres in* Naïve* groups.

Signals for pCREB, CREB, PSD 95, and vGlut1 were identified and measured by IMARIS software (version 7.6.5). For pCREB and CREB level quantification, four nonoverlapping Regions of Interest (ROIs, 45 × 45 *µ*m squares) were randomly selected from each image; IMARIS software was used to automatically count the number of pCREB or CREB positive spots in each ROI.

Signals for PSD 95 and vGlut1 were detected separately from ten nonoverlapping ROIs (10 × 10 *µ*m squares) from each image; ROIs were randomly selected avoiding DAPI-labeled cell bodies. IMARIS software was used for automatic detection and counting of PSD 95 and vGlut1 puncta and for identification of PSD 95/vGlut1 colocalizations as overlapping signals.

For all the experiments, ROIs were analyzed after establishing a detection threshold, which was kept constant within each measurement. All values were averaged per hemisphere (*Naïve*,* Contra*, and* Ipsi*).

### 2.6. Statistics

Group differences for values of pCREB and CREB expression and spine density were analyzed by means of two-way ANOVAs with genotype (wild type and mCREB) and experimental group (*Naïve*,* Ipsi*, and* Contra*) as between factors. Three-way ANOVA with genotype (wild type and mCREB), experimental groups (*Naïve*,* Ipsi*, and* Contra*), and dendritic category (apical and basal) was also used to compare spine density data among apical versus basal dendrites.

vGlut1 and PSD 95 signals and their colocalization signals were analyzed by means of separated one-way ANOVAs experimental group (*Naïve*,* Ipsi*, and* Contra*) as between factors. Where reported, differences between wild type* Naïve* and mCREB* Naïve* mice were estimated by one-way ANOVAs with genotype as between factors. Cumulative frequencies of spine head diameters were compared by Kolmogorov-Smirnov tests. Where necessary, Fisher LSD post hoc tests were used for pair comparisons.

## 3. Results

### 3.1. Unilateral Whisker Cut Associates with CREB Phosphorylation in* Contra* and* Ipsi* Barrel Cortex

To determine whether changes in whisker inputs induce the activation of the transcription factor CREB in related somatosensory cortex, we quantified the expression of total CREB (CREB) and phosphorylated CREB (pCREB) in barrel cortex of young adult mice unilaterally deprived of vibrissae. Whiskers were cut daily from PND 30 to PND 44 (Figure 1) in wild type and mCREB mice. Signals for CREB and pCREB expression were identified by immunofluorescent detection in brain slices containing barrel cortex of* Contra*,* Ipsi* to trimmed side and from* Naïve* mice of both genotypes.

**Figure 2:**
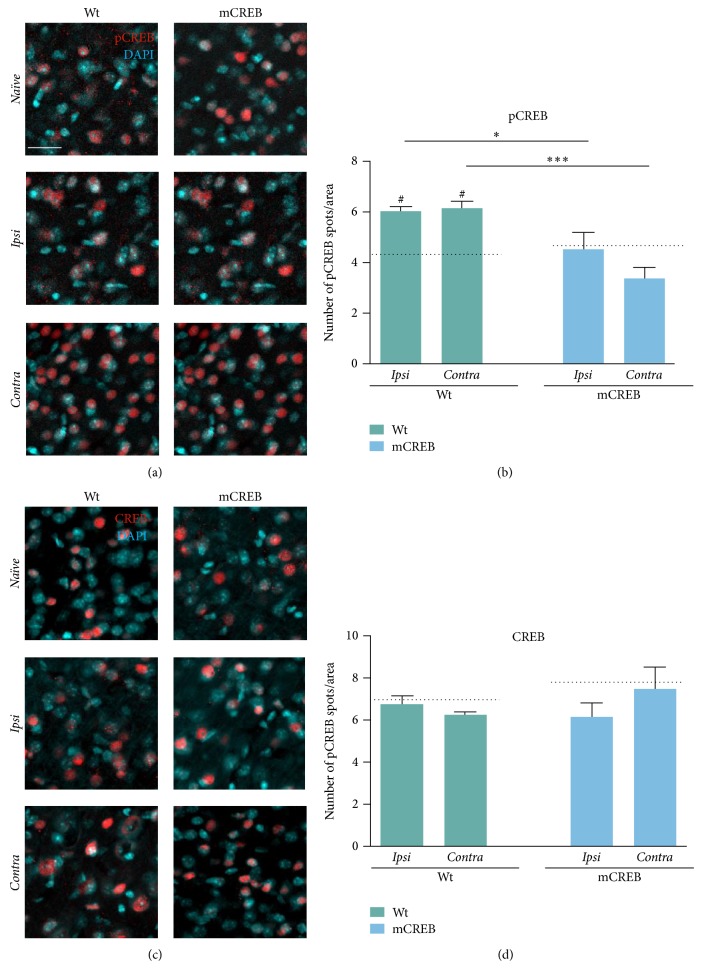
Whisker trimming associates with CREB phosphorylation in* Contra* and* Ipsi* barrel cortex without perturbing total CREB expression. Representative images of (a) phosphorylated CREB (pCREB, red) and (c) total CREB (CREB, red) expression in immunofluorescence-stained barrel cortex sections. Sections were counterstained with DAPI (blue). Scale bar 30 *µ*m. Histograms showing the average number of (b) pCREB and (d) total CREB positive spots per area from wild type (WT) and mCREB mice in* Naïve* and trimmed condition (*Naïve*,* Ipsi*, and* Contra*). Values are plotted as number of positive spots per ROI and expressed as mean ± s.e.m. Dotted lines indicate number of spots in barrel cortex of relative* Naïve* control mice. # < 0.05 (difference from relative* Naïve* controls); ∗∗∗ < 0.001; ∗ < 0.05 (difference between genotypes). *N*: 4 to 6 hemispheres for each group.

Results indicate that pCREB (Figures 2(a) and 2(b)) is differently expressed among the two genotypes (effect of genotype: *F*_1,26_ = 17.28, *p* < 0.001) upon trimming. Pair comparisons revealed that in wild type mice the number of pCREB positive neurons rises significantly in both* Ipsi* and* Contra* barrel cortex as compared to* Naïve* mice barrel cortex (wild type* Contra* versus* Naïve*, *p* < 0.05; wild type* Ipsi* versus* Naïve*, *p* < 0.05). Conversely, in mCREB mice, pCREB positive spots measured in the* Ipsi* and* Contra* barrel fields are unaltered as compared to* Naïve* (*P* > 0.05 for both comparisons).

As predictable, total CREB levels (Figures 2(c) and 2(d)) are comparable between wild type and mCREB mice in* Naïve* (*F*_1,10_ = 0.47, *p* = 0.5) condition and are unaltered upon whisker trimming in both* Ipsi* and* Contra* barrel cortex (genotype × condition: *F*_2,30_ = 2.30, *p* = 0.11).

Together, the above data indicate that prolonged whisker cut in wild type mice results in CREB activation upon phosphorylation in cortices* Contra* and* Ipsi* to the trimmed mystacial pad. This prompted us to investigate the role of CREB in rearrangements of synaptic circuit occurring in response to whisker cut.

### 3.2. Prolonged Unilateral Whisker Trimming Leads to a CREB-Mediated Increase of Dendritic Spines Number in the* Contra* Barrel Cortex

**Figure 3:**
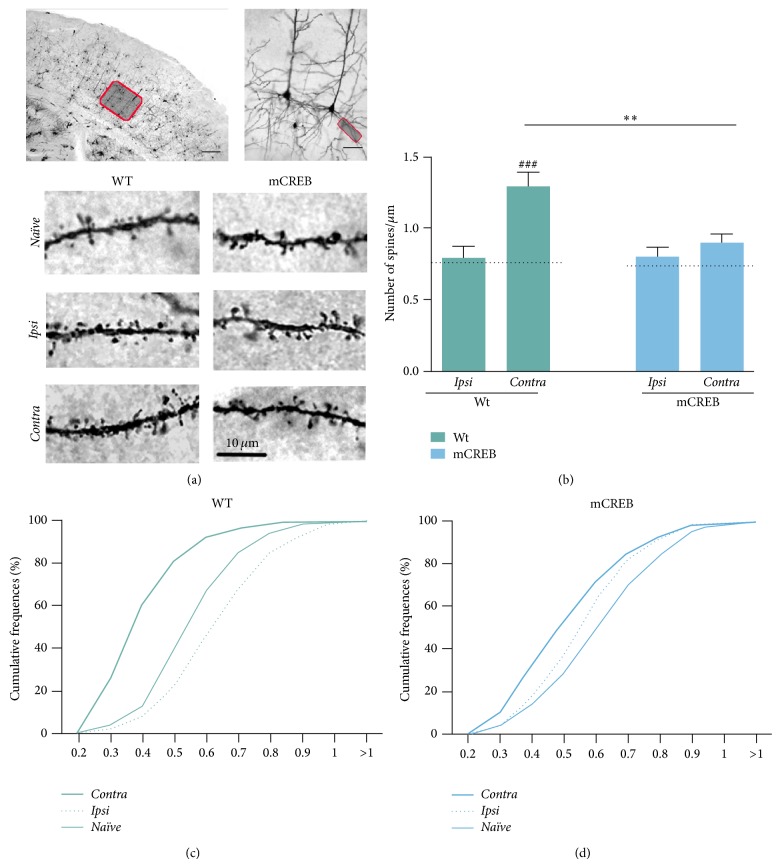
CREB is necessary for dendritic spine reorganization associated with whisker trimming. (a) Photomicrographs of Golgi-stained barrel cortex area (top-left, red square refers to layer V barrel cortex area; scale bar 250 *µ*m), representative barrel cortex neurons (top-right, red square refers to basal dendrites along layer V neurons; scale bar 50 *µ*m), and segments of basal dendrites (bottom, scale bar 10 *µ*m) from wild type (WT) and mCREB mice in* Naïve* and trimmed condition (*Naïve*,* Ipsi*, and* Contra*). (b) Histograms depicting dendritic spine density measured on basal dendrites of layer V pyramidal neurons in wild type (WT) and mCREB trimmed mice. Values are expressed as number of spines (mean ±  s.e.m.) per 1 *µ*m segment. Dotted line indicates average spine density in relative* Naïve* groups. ### < 0.001 (difference from relative* Naïve* controls); ∗∗ < 0.01 (difference between genotypes). ((c)-(d)) Cumulative frequencies relative to head diameter widths measured on dendritic spines of Wt (c) and mCREB mice (d). *N* = 6 mice for each genotype, 7/8 neurons for each mouse, about 600 spines for each experimental group.

Dendritic spines, the sites of excitatory synapses, are extremely dynamic structures and their modification in number or shape is an important index of synaptic plasticity occurring in response to external inputs. To investigate the role of CREB in trimming-induced spine changes, we measured dendritic spine density along apical and basal dendrites in layer V pyramidal neurons of Golgi-stained (Figure 3(a)) barrel cortices. Samples of* Contra* and* Ipsi* barrel cortices from trimmed mice and samples of barrel cortex from* Naïve* mice from the two genotypes were included in the analysis. First, we demonstrated that CREB downregulation does not affect the number of spines in barrel cortex of* Naïve* mice, as spine density (number of spines per unit length) is comparable between* Naïve* mCREB and wild type mice along both basal (Figures 3(a) and 3(b)) (*F*_1,25_ = 0.06413, *p* = 0.80) and apical dendrites (for details, see supplementary Figure  1 in Supplementary Material available online at http://dx.doi.org/10.1155/2015/651469). Rather, the trimming condition exerts a relevant effect on spine density in wild type but not mCREB mice. Along basal dendrites of wild type mice (Figure 3(b)), whisker trimming induces a significant increase of dendritic spines number in the* Contra* barrel cortex controlling the trimmed side as compared to* Ipsi* and* Naïve* barrel fields (interaction genotype × condition: *F*_2,68_ = 3.56, *p* < 0.05; post hoc comparisons: wild type* Contra* versus* Naïve*, *p* < 0.001; wild type* Contra* versus* Ipsi*, *p* < 0.01). Conversely, in mCREB mice, the number of spines along basal dendrites was unvaried upon trimming in both* Contra* and* Ipsi* as compared to* Naïve* barrel cortex (*P* > 0.05 for all comparisons). Together, these data indicate that whisker trimming results in a CREB-dependent increase of dendritic spines in* Contra* barrel cortex. An analogous trimming-related increase of dendritic spines was also reported along apical dendrites of Contra barrel cortex in wild type but not mCREB mice (see supplementary Figure  1 for details). Furthermore, we reported that the amount of spines number changes was comparable between apical and basal dendrites of mCREB and wild type mice in the three groups (genotype × condition × dendritic category: *F*_2,128_ = 0.30, *p* > 0.05; see Supplementary Figure  1 for details). Therefore, we restricted further analysis to basal dendrites only, which can be easily identified in immunofluorescence experiments below.

### 3.3. Prolonged Unilateral Whisker Trimming Associates with a CREB-Dependent Enlargement of Dendritic Spines in the* Ipsi* Barrel Cortex

Spines forming in response to external inputs are small and highly unstable protrusions that can either mature and stabilize into larger spines or shrink and undergo fast pruning. Therefore, classification of a spine based on its shape is an important index of its maturity and stability.

To investigate whether prolonged trimming affects maturation of spines, we classified barrel cortex spines based on the size of their heads. To this aim, we measured head width (spine head diameter, see detail in Figures 3(c) and 3(d)) on a subset of spines from* Ipsi* and* Contra* barrel cortex of wild type and mCREB mice undergoing whisker trimming and from barrel fields of* Naïve* mice.

Albeit the number of spines is comparable in the barrel cortex of wild type and mCREB mice under standard conditions, we found that spines in wild type* Naïve* mice appear thinner than spines in mCREB* Naïve* mice (average spine head width 0.54 *μ*m in wild type and 0.60 *μ*m in mCREB; Kolmogorov-Smirnov test: *p* < 0.001). In any case, in both genotypes, the majority of spines appear large and mushroom-like, as expected from mature stable spines.

To evaluate size variation induced by whisker trimming, spine head diameter values were then plotted in cumulative frequency graphs reported in Figures 3(c) and 3(d). In wild type mice, unilateral trimming results in an enhanced proportion of thin spines in the* Contra* barrel cortex (average spine head width of 0.38 *μ*m) and of large spines (average spine head width of 0.62 *μ*m) in the* Ipsi* barrel cortex as compared to relative* Naïve* controls. These effects are revealed by the left-shift of* Contra* group curve (Kolmogorov-Smirnov test: *p* < 0.001) and the right-shift of* Ipsi* group curve (Kolmogorov-Smirnov test: *p* < 0.001) with respect to the* Naïve* curve (Figure 3(c)). This set of data is consistent with the possibility that most spines in the* Contra* side are newly formed, as indicated from their increased number and thin shape. Rather, enlargement of existing spines is an index of their stabilization. Consistently, we found that the majority of spines in the* Ipsi* side appear to be mature, as indicated by their larger mushroom-like size. In stark contrast, in mCREB mice, trimming results in an enhanced proportion of thin spines in both the* Ipsi* (average spine head width of 0.55 *μ*m) and the* Contra* cortices (average spine head width of 0.50 *μ*m) as indicated by the left-shift of both curves (Figure 3(d)) with respect to* Naïve* controls (Kolmogorov-Smirnov test: *p* < 0.01 for both). This last result suggests that, in the absence of CREB regulation, unilateral whisker trimming favors spine shrinkage in barrel cortices controlling the whisker-deprived and the whisker-spared side.

### 3.4. The Formation of Synaptic Contacts Associated with Whisker Trimming in Barrel Cortex Requires CREB

**Figure 4:**
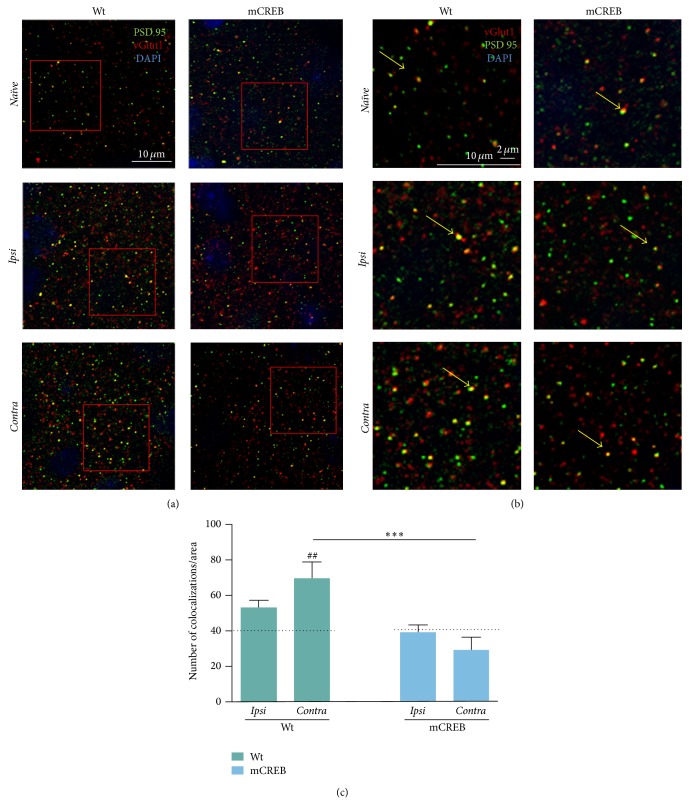
CREB function is necessary for whisker trimming-induced increase of synaptic contacts in* Contra* barrel cortex. (a) Representative images from wild type (WT) and mCREB mice in* Naïve* and trimmed conditions (*Naïve*,* Ipsi*, and* Contra*, scale bar 10 *μ*m). Sections were stained for the presynaptic marker vesicular glutamate transporter 1 (vGlut1) (Red), the postsynaptic marker PSD 95 (Green), and DAPI (blue). Red squares refer to areas magnified in (b). Colabelled puncta are shown in yellow; arrows mark examples of colaballed puncta (one example for each image). (c) Histogram showing the mean number of colocalizations of wild type (WT) and mCREB mice in trimmed condition. Dotted line indicates the average number of colocalizations in the relative* Naïve* group. Colocalization was plotted as number of overlapping signals (mean ± s.e.m.) per 100 *µ*m^2^. ## < 0.01 (difference from relative* Naïve*); ∗∗∗ < 0.001 (difference between genotypes). *N*: 5 to 6 hemispheres for each group.

The above results revealed that whisker cut shapes spines in the barrel cortex, but whether variations in number and morphology of dendritic spines associate with variations in the amount of excitatory inputs within the barrel field is still in question. To address this issue, we probed whether structural plasticity of spines was accompanied by variations in the number of excitatory synaptic inputs to layer V neurons [23]. To this aim we measured colocalization of pre- and postsynaptic markers by immunofluorescent detection in layer V barrel fields of the three experimental groups (*Ipsi*,* Contra*, and* Naïve*) from wild type and mCREB mice (Figure 4). As presynaptic marker, we used vesicular glutamate transporter 1 (vGlut1), the principal glutamate transporter in adult pyramidal neurons [24]; to label postsynaptic sites we used PSD 95, a major constituent of postsynaptic density [25].

Table 1 shows the amount of vGlut1 puncta, PSD 95 puncta, and their colocalization in each experimental group. Values of these three measurements are comparable between wild type and mCREB mice under* Naïve* conditions (vGlut1: *F*_1,10_ = 1.10, *p* = 0.3; PSD 95: *F*_1,10_ = 2.17, *p* = 0.18; vGlut1/PSD 95 colocalization: *F*_1,10_ = 0.06, *p* = 0.81).

Rather, unilateral whisker trimming exerts different effects on these three measurements in wild type and mutant mCREB mice. In wild type mice, trimming results in a significant increase of PSD 95 labelling (*F*_2,15_ = 6.18, *p* < 0.05) in* Contra* (*p* < 0.01) but not* Ipsi* (*p* > 0.05) hemisphere as compared to* Naïve*. Furthermore, trimming results in a significant increase of vGlut1 labelling (*F*_2,15_ = 6.01, *p* < 0.05) in both* Contra* (*p* < 0.01) and* Ipsi* (*p* < 0.05) hemispheres and in a significant increase of PSD 95/vGlut1 colocalization (*F*_2,15_ = 4.36, *p* < 0.05) in* Contra* hemisphere only (*Naïve* versus* Contra*: *p* < 0.01,* Naïve* versus* Ipsi*: *p* > 0.05).

In mCREB mice, trimming has no effect on any of the three measurements (vGlut1: (*F*_2,11_ = 2.28, *p* > 0.05), PSD 95: (*F*_2,11_ = 1.95, *p* > 0.05), and vGlut1/PSD 95 colocalization: (*F*_2,11_ = 1.45, *p* > 0.05)), albeit we detected a nonsignificant decrease of PSD 95 in* Contra* hemisphere (*Naïve* versus* Contra*: *p* = 0.07) and a nonsignificant increase of vGlut1 in* Ipsi* hemisphere (*Naïve* versus* Ipsi*: *p* = 0.08).

## 4. Discussion

The transcription factor CREB is an important player in activity-dependent neuronal plasticity, and several evidences reported the activation of CREB-mediated gene transcription in relation to experience-mediated plasticity within neocortical regions [26–31]. Here we show that variations in sensory inputs induce CREB activation in barrel fields of somatosensory cortex and that CREB activation in turn participates in related long-lasting structural rearrangements of layer V barrel cortex synapses.

We stimulated changes in sensory inputs through whisker manipulation in transgenic mice expressing the dominant negative mCREB mutation [19]. mCREB is a phosphorylation-defective form of CREB that can bind and occupy CRE sites, thus repressing endogenous CREB function and interfering with the expression of genes that have CRE sequence in their promoters [19].

We first show that, in mice under baseline condition, CREB downregulation slightly modifies the size of dendritic spines in barrel cortex. In mCREB mice left undisturbed in their home cage (*Naïve*), spines in layer V barrel cortex appear slightly larger as compared to wild type under the same condition (Figures 3(c) and 3(d)), although the number of spines is comparable among the two groups (Figure 3(b)). In cortical regions, spines are extremely dynamic structures that undergo ongoing and input-independent modifications in shape and/or size [32]. Therefore, our data above suggests that CREB may play a role in this ongoing modelling of dendritic spines. Regardless of spine size differences, synaptic function is intact in mCREB mice under* Naïve* condition, as demonstrated from the following evidences: (i) maturity and stability of dendritic spines are comparable between mCREB and wild type mice, as indicated by the typical mushroom-like shape of spines in both groups (Figure 3(a)); (ii) the number of putative synaptic contacts, as identified by presynaptic vGlut1 and postsynaptic PSD 95 colocalizations, is similar between mCREB and wild type mice (Figure 4). Together, these evidences confirm our [14] and others' [33] studies demonstrating that CREB is not required for baseline synaptic function.

Rather, evidences presented here indicate that CREB function is necessary for plasticity events associated with environmental stimulation of sensory inputs. Two main results emerged from our study. The first concerns the effects that prolonged whisker deprivation has on the barrel field lying in the hemisphere contralateral to the trimmed side (*Contra*). The second refers to the effects of spared sensory inputs arriving from the nontrimmed mystacial pad which are detectable in the hemisphere ipsilateral to the trimmed side (*Ipsi*).

First, we provide evidences that prolonged whisker deprivation induces CREB phosphorylation in the* Contra* barrel cortex (Figure 2). This data is in line with evidences reporting CRE-mediated gene transcription in adult barrel cortex after whisker cut [31] and suggests that CREB can be involved in neuronal rearrangements occurring in association with sensory deprivation. Consistent with this possibility, we here probed that CREB function is required for the formation of new dendritic spines in barrel cortex in response to prolonged whisker deprivation (Figure 3(b)). A pivotal two-photon microscopy study performed on living mice by Gan's group [1] previously demonstrated that prolonged trimming blocks ongoing baseline spine elimination in layer V barrel cortex neurons but does not affect the process of spine formation along the same dendrites; the combination of these two events results in net enhancement in the number of spines. Consistent with this evidence, we show that, in wild type mice, unilateral whisker trimming associates with increased number of spines (Figure 3(b) and supplementary Figure  1) in dendrites of layer V* Contra* barrel cortex; furthermore, that the vast majority of those spines display the thin shape (Figure 3(c)) is indicative of a recent formation [34].

In the* Contra* barrel cortex of mCREB mice, the number of spines remains unvaried (Figure 3(b)); however, those spines appear slightly reduced in size with respect to spines from relative* Naïve* controls (Figure 3(d)), suggesting that unilateral trimming leads to the shrinkage of existing spines. Together, the above data indicate that CREB is required for both the formation of new dendritic spines and the maintenance of old preexisting ones.

Our results are in line with previous studies probing the importance of CREB in the modulation of spines number and morphology. In cultured hippocampal neurons, increased CREB phosphorylation favors spinogenesis [11] while decreasing CREB function results in block of spine formation [10].* In vivo*, viral injection of a constitutively active form of CREB (caCREB) in the CA1 hippocampal region leads to an increase of spines along pyramidal neurons in this region [9]. Also, we previously reported [14] that the expression of the mCREB mutation in adult mice inhibits dendritic spine growth that occurs in the hippocampus in association with the formation of a spatial memory.

It has been proposed [35] that thin, filopodia-like spines are formed independently of synaptic inputs and then search for a presynaptic contact. Consistent with this possibility, spine growth has been shown to precede synapse formation in adult neocortex [4]. We therefore asked whether new spines formed in association with whisker input deprivation support active synapses or rather new thin spines lack synaptic contacts. Our results indicate that in wild type mice the formation of new dendritic spines in the* Contra* barrel fields cooccurs with enhanced proportion of pre- and postsynaptic markers (Table 1) and of putative synaptic contacts (Table 1 and Figure 4). This evidence strongly supports the possibility that new spines formed in association with whisker trimming hold new synaptic contacts and account for changes in circuit connectivity.

In the* Contra* barrel cortex of mCREB mice, the absence of putative new synaptic contacts (Figure 4) parallels the lack of new spines formation, indicating that CREB function is required for both of these plastic events associated with whisker trimming. More in detail, we show that absence of new synaptic contacts depends on both a pre- and a postsynaptic defect: first, vGlut1 levels do not change in relation to whisker trimming (Table 1). This data confirms previous evidences showing that CREB function is required for presynaptic events associated with synaptic plasticity [31]. Second, PSD 95 levels tend to drop down (Table 1) upon whisker trimming, indicating that CREB downregulation impacts the production of PSD 95 and possibly related synaptic trafficking of glutamatergic receptors [20]. This second evidence is consistent with the reported shrinkage of dendritic spines in this group and confirms that CREB favors molecular events associated with dendritic spines stabilization [14, 20].

The second set of results of our study relates to sensory stimulation coming from the nontrimmed mystacial pad, which is detectable in the hemisphere ipsilateral to the trimmed side (*Ipsi*). Movement of vibrissae constitutes the main sensory input for a rodent exploring the surrounding environment. Previous studies on whisker deprivation reported both potentiation of responses from spared vibrissae [27] and expansion of the cortical area responding to intact whiskers [36]. These evidences indicate that inputs from spared whiskers can compensate for the lack of stimuli from the nearby mystacial region and then contribute to enhancing plasticity, a process that resembles the well-described shift in ocular dominance toward the nondeprived eye upon eyelid closure [37]. This compensatory plasticity requires CREB, as demonstrated by evidences showing that CRE-mediated gene transcription associates with potentiation of spared whisker responses upon trimming of all-but-one whiskers [31]. In line with those evidences, we here probed that CREB phosphorylation is induced in* Ipsi* barrel cortex in response to prolonged trimming (Figures 2(a) and 2(b)) and we asked whether CREB is involved in synaptic and spine rearrangements occurring in the barrel cortex that controls spared whiskers.

We report that spines along layer V neurons in* Ipsi* barrel cortex of wild type mice are significantly enlarged as compared to relative* Naïve* controls (Figure 3(c)). This spine enlargement requires CREB, since, in mCREB mice, trimming induces the shrinkage of spines in* Ipsi* cortex (Figure 3(d)).

In wild type mice, spine enlargement associates with a trend toward increase of putative synaptic contacts (Figure 4), possibly due to the spreading of presynaptic vGlut1 (Table 1). This suggests that new synaptic contacts may form between newly available vGlut1 and previously existing PSD 95 content in dendritic spines, a possibility supported by the evidences that not all available vGlut1 and PSD 95 form synaptic contacts [38], as we reported in* Naïve* wild type mice (Table 1).

In* Ipsi* cortex of mCREB mice, vGlut1/PSD 95 colocalizations are unaltered as compared to relative* Naïve* controls; this data indicates that CREB-mediated formation of new synaptic contacts may be involved in the stabilization of preexisting dendritic spines.

Collectively, our findings indicate that CREB plays a key role in rearrangements of dendritic spine and synaptic circuits that account for barrel cortex connectivity in response to changes in the environment. Furthermore, our study opens important questions concerning the relation between availability of pre- or postsynaptic content and the amount of new spines formation.

In summary, the study described here points to CREB as a key player in structural rearrangements of circuit connectivity in response to changes of sensory stimulation. The role of CREB in favoring spine formation or enlargement and related synaptic circuit modulation is important in view of pathological conditions such as Alzheimer's Disease or Fragile × syndrome in which alterations of spines and synaptic circuits relate to impaired cognition [7]. A possibility emerging from our study is that modulation of the CREB pathway in those pathological conditions might restore spine structure; this in turn might contrast the effects of altered synaptic connectivity and related cognitive impairment.

## Supplementary Material

We confirmed that changes in the number of dendritic spines along apical dendrites of Layer V pyramidal neurons were similar to the ones reported along basal dendrites of the same neurons.
CREB down-regulation does not affect the number of spines in barrel cortex of *Naïve* mice, as spine density (number of spines per unit length) is comparable between *Naïve* mCREB and wild type mice along apical dendrites (P>0.05). Trimming condition results in enhanced number of spines in wild type but not mCREB mice (interaction genotype x condition: F_2_, _60_=5.7532, p<0.01). Wild type mice: significant increase of dendritic spine number along apical dendrites of pyramidal neurons in the *Contra* barrel cortex as compared to *Ipsi* and *Naïve* (post-hoc comparisons: *Contra* vs *Naïve* p<0.001; *Contra* vs *Ipsi* p<0.001). In *Ipsi* barrel cortex the number of spines was not affected by trimming condition (*Ipsi* vs *Naïve* p>0.05). mCREB mice: number of spines was unvaried upon trimming in both *Contra* and *Ipsi* as compared to *Naïve* barrel cortex (p > 0.05 for all comparisons).
No differences between number of dendritic spines along apical dendrites versus basal dendrites were reported (genotype x condition x dendritic category: F_2,128_=0.30, p>0.05; Post hoc: p>0.05 for all *Naïve*, *Contra* and *Ipsi* apical dendrites vs basal dendrites comparisons in both genotypes).

## Figures and Tables

**Table 1:** Number of vGlut1 and PSD 95 positive puncta and number of colocalizations in the six experimental groups.

		PSD 95		vGlut1		Colocalization	
		Mean	s.e.m.	Mean	s.e.m.	Mean	s.e.m.
WT	*Naïve *	102.1	8.3	82.0	7.5	40.0	7.8
	*Ipsi *	109.2	10.6	130.0^∗^	18.4	53.0	4.2
	*Contra *	151.7^∗∗^	13.7	132.0^∗∗^	13.9	69.5^∗∗^	9.3

mCREB	*Naïve *	83.4	9.9	93.2	13.9	42.4	6.6
	*Ipsi *	71.4	10.0	122.6	15.6	39.0	4.3
	*Contra *	54.3	7.1	96.0	12.9	29.0	7.4

Values were mean ± s.e.m. ^∗^<0.05; ^∗∗^<0.01 (difference from relative *Naïve* controls).
