# Correction to “CREB Regulates Experience‐Dependent Spine Formation and Enlargement in Mouse Barrel Cortex”

**DOI:** 10.1155/np/9896025

**Published:** 2026-05-18

A. Pignataro, A. Borreca, M. Ammassari‐Teule, and S. Middei, “CREB Regulates Experience‐Dependent Spine Formation and Enlargement in Mouse Barrel Cortex,” *Neural Plasticity* 2015, no. 1 (2015): 651469, https://doi.org/10.1155/2015/651469.

In the above article, the panels depicting “*Ipsi*” and “*Contra*” in Figure 2 (a) had been mistakenly duplicated across both conditions during typesetting. The colour key had also been reversed.

When addressing these errors, it was also noted that the left middle panel (“Wt – *Ipsi*”) and right bottom panel (“mCREB – *Contra*”) had been positioned incorrectly during figure preparation. The correct Figure 2 is as follows:

Figure 2Whisker trimming associates with CREB phosphorylation in *Contra* and *Ipsi* barrel cortex without perturbing total CREB expression. Representative images of (a) phosphorylated CREB (pCREB, red) and (c) total CREB (CREB, red) expression in immunofluorescence‐stained barrel cortex sections. Sections were counterstained with DAPI (blue). Scale bar 30 *μ*m. Histograms showing the average number of (b) pCREB and (d) total CREB positive spots per area from wild type (WT) and mCREB mice in *Naïve* and trimmed condition (*Naïve*, *Ipsi*, and *Contra*). Values are plotted as number of positive spots per ROI and expressed as mean ± s.e.m. Dotted lines indicate number of spots in barrel cortex of relative *Naïve* control mice. # < 0.05 (difference from relative *Naïve* controls); ∗∗∗ < 0.001; ∗< 0.05 (difference between genotypes). *N*: 4–6 hemispheres for each group.

**Figure:**
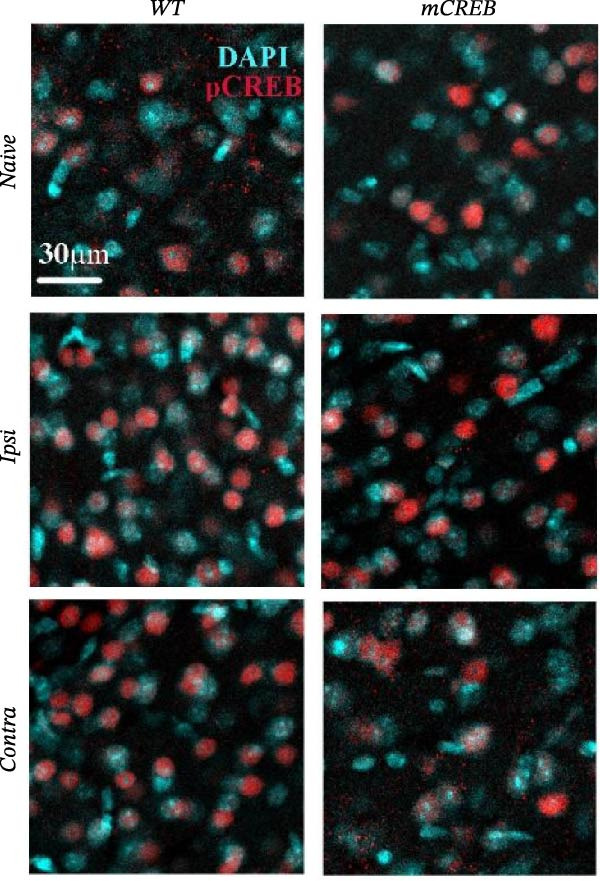
(a)

**Figure:**
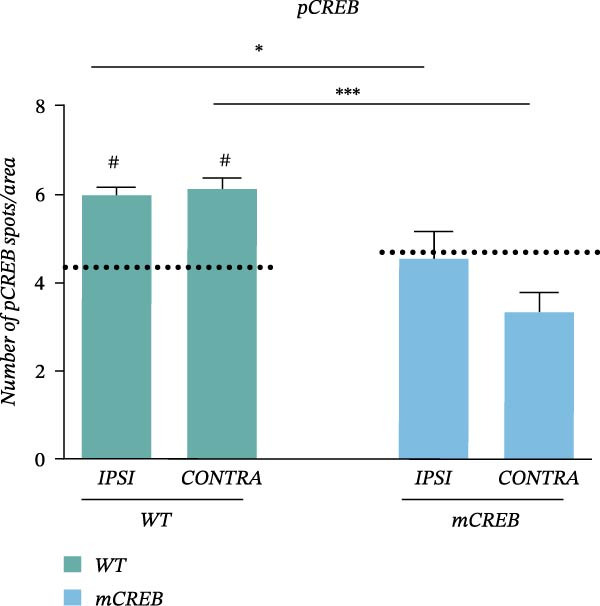
(b)

**Figure:**
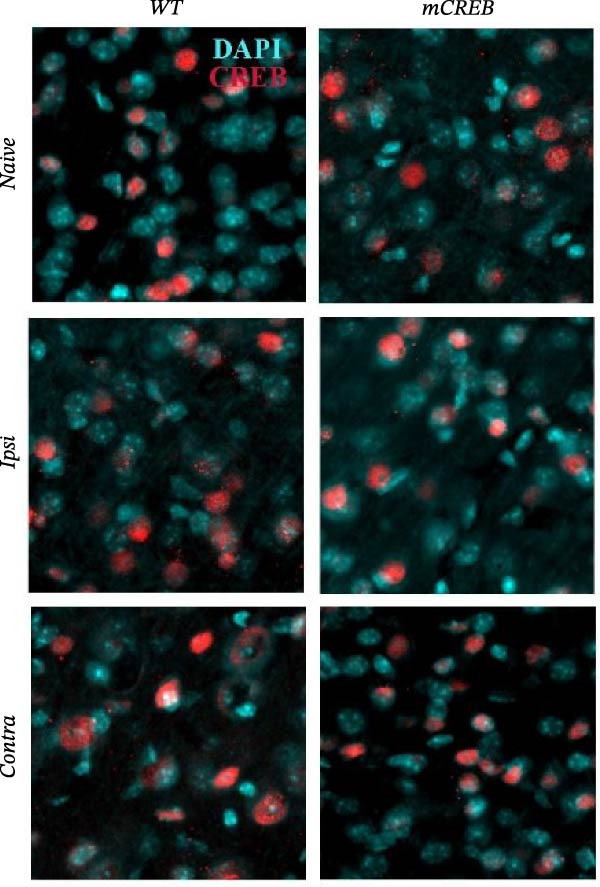
(c)

**Figure:**
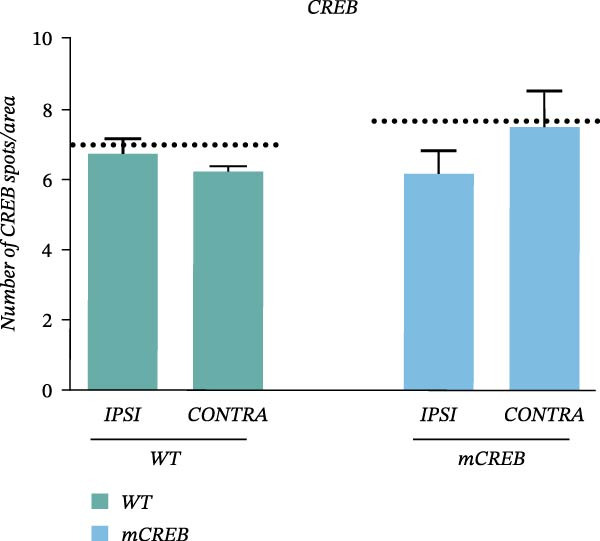
(d)

We apologize for this error.

