# Supplementary material for: CREB Regulates Experience-Dependent Spine Formation and Enlargement in Mouse Barrel Cortex
Source: Neural Plast. 2015 May 5;2015:651469. doi: 10.1155/2015/651469 (PMC4436461; doi:10.1155/2015/651469)
Supplement: Supplementary file 1 — We confirmed that changes in the number of dendritic spines along apical dendrites of Layer V pyramidal neurons were similar to the ones reported along basal dendrites of the same neurons. CREB down-regulation does not affect the number of spines in barrel cortex of Naïve mice, as spine density (number of spines per unit length) is comparable between Naïve mCREB and wild type mice along apical dendrites (P>0.05). Trimming condition results in enhanced number of spines in wild type but not mCREB mice (interaction genotype x condition: F2, 60=5.7532, p<0.01). Wild type mice: significant increase of dendritic spine number along apical dendrites of pyramidal neurons in the Contra barrel cortex as compared to Ipsi and Naïve (post-hoc comparisons: Contra vs Naïve p<0.001; Contra vs Ipsi p<0.001). In Ipsi barrel cortex the number of spines was not affected by trimming condition (Ipsi vs Naïve p>0.05). mCREB mice: number of spines was unvaried upon trimming in both Contra and Ipsi as compared to Naïve barrel cortex (p > 0.05 for all comparisons). No differences between number of dendritic spines along apical dendrites versus basal dendrites were reported (genotype x condition x dendritic category: F2,128=0.30, p>0.05; Post hoc: p>0.05 for all Naïve, Contra and Ipsi apical dendrites vs basal dendrites comparisons in both genotypes). [file 651469.f1.pdf]

Supplementary figure 1

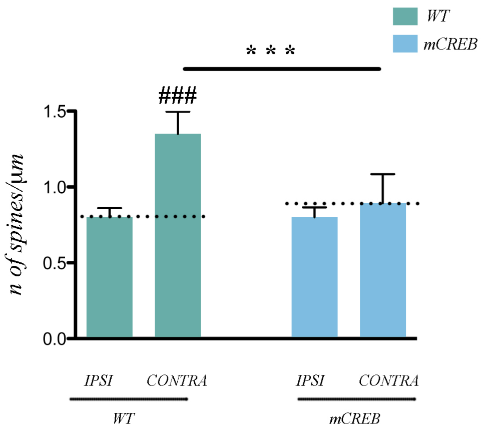

Supplementary figure 1

Histograms showing dendritic spine density along apical dendrites of layer V pyramidal neurons in wild type (WT) and mCREB mice.

Trimming condition results in enhanced number of spines in wild type but not mCREB mice (interaction genotype x condition: $F_{2, 60}=5.7532$, $p<0.01$). Wild type mice: significant increase of dendritic spine number along apical dendrites of pyramidal neurons in the *Contra* barrel cortex as compared to *Ipsi* and *Naïve* (post-hoc comparisons: *Contra* vs *Naïve* $p<0.001$; *Contra* vs *Ipsi* $p<0.001$). In *Ipsi* barrel cortex the number of spines was not affected by trimming condition (*Ipsi* vs *Naïve* $p>0.05$). mCREB mice: number of spines was unvaried upon trimming in both *Contra* and *Ipsi* as compared to *Naïve* barrel cortex ($p > 0.05$ for all comparisons). Values are expressed as number of spines (mean±s.e.m) per 1 μm segment. Dotted line indicates average spine density in relative naïve groups. ###< 0.001 (difference from relative naïve); ***<0.001 (difference between genotypes).

No differences between number of dendritic spines along apical dendrites versus basal dendrites were reported (genotype x condition x dendritic category: $F_{2,128}=0.30$, $p>0.05$; Post hocs: $p>0.05$ for all *Naïve, Contra* and *Ipsi* apical dendrites vs basal dendrites comparisons in both genotypes).
